# The effect of green tea extract supplementation on exercise-induced oxidative stress parameters in male sprinters

**DOI:** 10.1007/s00394-014-0757-1

**Published:** 2014-08-14

**Authors:** Ewa Jówko, Barbara Długołęcka, Beata Makaruk, Igor Cieśliński

**Affiliations:** 1 Department of Physiology and Biochemistry, Faculty of Physical Education and Sport in Biala Podlaska, Jozef Pilsudski University of Physical Education in Warsaw, Akademicka 2, 21-500 Biala Podlaska, Poland; 2 Faculty of Physical Education and Sport in Biala Podlaska, Jozef Pilsudski University of Physical Education in Warsaw, Akademicka 2, 21-500 Biala Podlaska, Poland

**Keywords:** Green tea extract, Oxidative stress, Sprinters, Repeated sprint test

## Abstract

**Background:**

Although research suggests that antioxidant supplementation can protect against exercise-induced muscle damage and oxidative stress, also delayed post-exercise muscle recovery and hindered adaptation to training were reported in the supplemented athletes.

**Purpose:**

The purpose of the study was to evaluate the effects of green tea extract (GTE) supplementation on selected blood markers of oxidative stress and muscle damage in sprinters during preparatory phase of their training cycle.

**Methods:**

Sixteen sprinters participated in a double-blind, randomized, placebo (PL)-controlled crossover study, including two 4-week treatment periods with PL and GTE (980 mg polyphenols daily). The sprinters performed two repeated cycle sprint tests (RST; 4 × 15 s, with 1-min rest intervals), after PL and GTE supplementation. Blood was sampled before (at rest), 5 min after RST, and after the 24-h recovery. The activities of superoxide dismutase (SOD) and glutathione peroxidase were measured in erythrocytes, and total polyphenols, total antioxidant capacity (TAC), uric acid (UA), albumin (AL), malondialdehyde (MDA), and creatine kinase (CK) were determined in blood plasma.

**Results:**

Repeated cycle sprint test performed after PL induced an increase in MDA, TAC, and SOD. Moreover, an increase in UA, AL, and CK was observed after RST irrespective of experimental conditions (PL, GTE). Supplementation with GTE caused an increase in total polyphenols and TAC at rest, and a decrease in MDA and SOD after RST. No significant changes in sprint performance were noted after GTE, as compared to PL.

**Conclusions:**

Supplementation with GTE prevents oxidative stress induced by RST in sprinters. Furthermore, GTE supplementation does not seem to hinder training adaptation in antioxidant enzyme system. On the other hand, neither prevention of exercise-induced muscle damage, nor an improvement in sprint performance is noted after GTE administration.

## Introduction

Both strenuous long-term and short-term anaerobic exercises were reported to enhance the generation of reactive oxygen species (ROS) by cells, which may lead to oxidative stress and cellular damage [1, 2]. On the other hand, ROS not only cause cellular damage, but also play an important role in modulation of cell signaling involved in training adaptations at the cellular level [3, 4]. One of these adaptations is the activation of redox-sensitive signal transduction pathway and enhancement in gene expression of antioxidant enzymes [4]. Training-induced up-regulation of endogenous antioxidants has been established to reduce the risk of cellular injury during acute exercise [1]. However, despite having increased superoxide dismutase (SOD) activity, athletes exhibit decreased total antioxidant status of plasma and elevated markers of lipid peroxidation and muscle damage as compared to sedentary subjects [5].

There is emerging evidence suggesting that ROS play an important physiological role in supporting the recovery process and protecting the cells from future damage [6]. Several studies revealed that antioxidant supplements may hinder the beneficial cell adaptations to exercise [7–9]. Moreover, delayed muscle recovery after acute exercise, as a result of 4-week antioxidant supplementation, was also reported [10]. However, these observations regarding the impaired training adaptation after antioxidant supplementation were not confirmed by other studies [11, 12]. Thus, the practice of taking antioxidants before exercise is recently a subject of ongoing debate and perhaps should be re-evaluated [13, 14].

Tea catechins, polyphenols with flavonoid structure, belong to chemical substances with strong antioxidant properties [15]. In our study of previously untrained men [16], the 4-week green tea extract (GTE) supplementation enhanced the total antioxidant potential of plasma and prevented oxidative damage induced by both short-term muscular endurance test and 4-week strength training. In another study [17], the protective effect of green tea beverages on oxidative stress and muscular damage parameters was also observed in weight-trained men. However, there is no published research on the effects of GTE supplementation on oxidative stress and muscle damage parameters in trained sprinters. It has been suggested that increased production of ROS during short-term, high-intensity exercise is mostly caused by the activation of xanthine oxidase (XO) pathway [18, 19]. Increases in both serum XO activity and serum concentration of uric acid (UA), i.e., the end product of XO pathway, have been reported after repeated sprint exercises as support of that thesis [20]. Correspondingly, the inhibition of XO (by allopurinol) has been reported to protect against cell damage induced by exhaustive exercise in humans [21]. On the other hand, when ROS production was prevented in rats, up-regulated expression of important enzymes associated with cell defense (like SOD) and adaptation to exercise were also abolished [22, 23]. Thus, it is interesting to investigate whether GTE supplementation can be beneficial for sprint-trained athletes during their habitual training schedule. The results of previous in vitro studies suggest that, apart from direct scavenging of ROS and chelating with metal ions [24], the antioxidant activity of tea catechins results from the inhibition of XO activity [25].

The aim of this study was to evaluate the effects of 4-week GTE supplementation on selected blood markers of oxidative stress [total antioxidant capacity (TAC), UA, albumin (AL), malondialdehyde (MDA), SOD, and glutathione peroxidase (GPx)] and muscle cell damage [creatine kinase (CK)] in male sprinters during preparatory phase of their yearly training cycle.

## Materials and methods

### Subjects

Sixteen male sprinters from a university sport club (aged 21.6 ± 1.5 years; body weight 76.9 ± 6.4 kg; height 180.5 ± 6.2 cm; BMI 23.5 ± 1.1 kg/m^2^; % body fat 11.8 ± 2.5 %; training experience 4.4 ± 1.4 years), all students of physical education, volunteered for the study. The exclusion criteria were the use of tobacco products, alcohol consumption, recent surgery or illness, and use of any medicine or dietary supplements in 4 weeks prior to the study. The protocol of the study was approved by the local Bioethical Committee at the Academy of Physical Education in Warsaw, and the research was conducted in accordance with the principles stated in the Declaration of Helsinki.

### Experimental procedures

The double-blind, randomized, placebo (PL)-controlled crossover study was conducted during preparatory phase of yearly training cycle (after transition period). The two 4-week treatment periods (during which half of the subjects received GTE and the other half PL, and vice versa) were separated by a 4-week washout period. The duration of the washout period was selected based on the results of one previous study [26], in which 6-week supplementation with 800 mg catechins daily was used. In that study, plasma catechin concentration returned to its baseline level after at least 2 weeks of washout period.

Both GTE and PL were administered in the form of dark gelatin capsules (Olimp Labs, Dębica, Poland), identical in appearance (i.e., size, shape, and color); the same dosage regimen was used (two capsules twice a day). One GTP capsule contained 250 mg of standardized GTE (245 mg polyphenols, including 200 mg catechins, among them 137 mg epigallocatechin-3-galate) and additional substances (maltodextrin, microcrystalline cellulose, and magnesium stearate). Therefore, each participant was administered 980 mg polyphenols daily. PL capsules contained microcrystalline cellulose, magnesium stearate, and maltodextrin instead of GTP. The compliance was measured by capsule counting. The participants who returned no more than 15 % of their capsule dose were classified as “compliant”.

At the end of each of the two 4-week treatment periods, the sprinters performed a repeated cycle sprint test (RST) on a cycle ergometer (Ergomedic 839E, Monark, Sweden). The test consisted of four consecutive 15-s bouts (4 × 15 s), each of them with base set according to the Wingate procedure and separated by 1-min rest intervals. The subjects were asked to cycle for 15 s, as fast as possible, against a constant load (75 g/kg body weight). The test was preceded by a 5-min warm-up with submaximal load, until the heart rate reached 130–150 beats/min (Polar, Finland). The following exercise parameters were registered (Multi CykloErgometr MCE version 5.1) during each of four 15-s bouts: peak power (W/kg), mean power (W/kg), total work output (J/kg), and fatigue index (%). The tests were performed in the morning following 12-h overnight fast, at air temperature between 19 and 21 °C and with 40–60 % relative humidity. The subjects were instructed to not perform hard physical training for 48 h and avoid drinking tea and caffeinated beverages within 24 h prior to each of the RSTs. The subjects were asked to refrain from any dietary supplements for the duration of the study.

### Dietary intake

The participants were asked to not modify their diet for the duration of the study, except for refraining from consuming any products containing green tea and limiting the intake of caffeine-containing drinks to one cup per day. Moreover, they were asked to maintain a similar diet for both treatment periods. During both the first and the second treatment periods (during 7 days preceding each RST), the participants filled out a 3-day dietary record (covering 2 week days and 1 day of the week end). The amount of ingested foods was estimated with an aid of dedicated picture book [27]. The daily dietary intake of vitamins C and E, beta-carotene, and selenium was calculated using Dietus software (based on national food tables) [28].

### Blood sampling

Capillary blood samples were taken from a finger pulp, before the test and 3 min after completing the test. Venous blood samples were drawn into heparinized test tubes from the ulnar vein before completing the test (at rest), 5 min after completing the test, and following the 24-h recovery period. The venous blood was centrifuged (for 10 min at 3,000×*g* at a temperature of 4 °C) to separate erythrocytes from plasma. Subsequently, erythrocytes were washed three times with a cold isotonic saline solution. Both erythrocytes and plasma were frozen and stored at −80 °C until analysis.

### Laboratory analysis

Capillary blood was assayed for the concentration of lactate, as well as for the parameters of acid–base equilibrium. The concentration of lactate was determined with a diagnostic cuvette kit (Dr. Lange, catalogue no. LKM 140, Germany) in a Miniphotometer Plus LP 20 (Hach Lange, Germany). An automated analyzer (OMNI-C analyzer, Roche Diagnostics, Austria) was used to measure the acid–base balance parameters in capillary blood: pH value, base excess, and anion gap, as well as hematocrit. According to the manufacturer, the intra-assay coefficients of variation for pH and hematocrit measurements were below 2.0 and 3.0 %, respectively.

Plasma concentrations of total polyphenols, UA, AL, and MDA were determined, along with TAC and the activity of CK. Erythrocytes were analyzed for the activity of SOD and GPx.

Analysis of total plasma polyphenols was performed using the Folin–Ciocatleau method, as described by Maskarinec et al. [29]. Gallic acid in ethanol served as the standard solution, and the results for total polyphenols were given as µg/ml gallic acid equivalents (GE). The average intra-assay coefficient of variation (calculated for ten duplicated samples) was 7.8 %.

The total antioxidant capacity of plasma (TAC) to scavenge ABTS radicals was measured by a chromogenic method with commercially available kit (Cat. No. NX 2332, Randox, Crumlin, UK). Antioxidant capacity of samples was expressed as millimoles per liter of Trolox equivalents (6-hydroxy-2,5,7,8-tetramethylchroman-2-carboxylic acid). The average intra-assay coefficient of variation (calculated for ten duplicated samples) was 4.9 %.

Plasma UA and AL concentrations were determined with commercially available kits (Cat. No. K6580-200 and A6502-100, respectively; Alpha Diagnostics, USA). The average intra-assay coefficients of variation (calculated for ten duplicated samples) for UA and AL were 6.1 and 4.3 %, respectively.

The SOD and GPx activities in erythrocytes were determined with commercially available kits (RANSOD Cat. No. SD 125 and RANSEL Cat. No. RS 505, respectively; Randox, Crumlin, UK). The antioxidant enzyme activities were measured at 37 °C and expressed in U/g Hb. Hemoglobin was assessed by a standard cyanmethemoglobin method, using a diagnostic kit (HG 1539; Randox, Crumlin, UK). The average intra-assay coefficient of variation (calculated for ten duplicated samples) for SOD, GPx, and Hb was 3.8, 6.9, and 2.9 %, respectively.

Plasma MDA levels were determined with a commercially available kit (LPO-586, OXIS Internatl., Portland, OR). Plasma CK activity was determined with the use of a diagnostic kit (Cat. No. C6512-100, Alpha Diagnostics, USA). The average intra-assay coefficient of variation (calculated for ten duplicated samples) for MDA and CK was 7.5 and 8.3 %, respectively. Due to the decreased plasma volume following exercise [30], plasma volume was corrected for post-exercise changes by considering the changes in hematocrit according to the Dill and Costill method [31].

### Statistical analysis

Statistical analysis was performed with Statistica version 6.0 software package. Paired *t* test was used to compare the results of the dietary survey and RST, and resting total plasma polyphenols levels documented after two 4-week treatment periods. The data regarding biochemical parameters were analyzed using 2 (treatment: PL and GTE) × 3 (time points: at rest, 5 min, and 24 h after completion of the test) factorial (two-way) ANOVA. If a significant time and treatment interaction was detected, the data within each treatment (at rest, 5 min post-exercise, and after 24-h of recovery) were analyzed and compared between treatments (PL and GTE) using the Bonferroni corrected paired *t* test. The normal distribution of all dependent variables was confirmed with the Shapiro–Wilk test and visual inspection (quantile distribution plots). All values were reported as mean ± SEM. The level of statistical significance was set at *p* < 0.05.

## Results

Mean daily energy intake and intakes of vitamins C and E, beta-carotene, and selenium in daily food rations of participants are presented in Table 1. No significant differences were found between PL and GTE treatments for any of the studied parameters. The diet was adequate in terms of the Polish Nutrition Society recommendations for physically active men [32]. The percentages of compliant were 100 % for both GTE and PL (compliance rates for GTE and PL capsules were 99 and 98 %, respectively). We did not document any dropouts or adverse effects of the supplementation.

**Table 1:** Daily intake of energy, vitamins C and E, beta-carotene, and selenium in sprinters (*n* = 16) after 4-week supplementation with placebo (PL) or green tea extract (GTE)

	PL	GTE
Energy (kcal)	4,072 ± 120	3,911 ± 105
Vitamin C (mg)	99 ± 3	95 ± 2
Vitamin E (mg)^a^	9.6 ± 0.3	9.1 ± 0.6
Beta-carotene (µg)	3,715 ± 105	3,749 ± 99
Selenium (µg)	91 ± 10	89 ± 10

Values are presented as mean ± SEM. No significant differences were documented between treatments (paired *t* test)

^a^mg tocopherol equivalents

After both GTE and PL supplementations, the RST induced a significant increase in plasma lactate concentration (Table 2) and caused the shift of acid–base balance toward metabolic acidosis (a drop in blood pH and an increase in base deficit and anion gap). The time effect (*p* < 0.0001) was observed in case of all the above-mentioned parameters.

**Table 2:** Changes in blood indices of acid–base balance and plasma lactate concentration induced by the repeated sprint test (4 × 15 s) in sprinters (*n* = 16) after 4-week supplementation with placebo (PL) or green tea extract (GTE)

	PL		GTE	
	Rest	3 min	Rest	3 min
pH	7.41 ± 0.004	7.18 ± 0.01***	7.42 ± 0.006	7.17 ± 0.01***
Base excess (mmol/L)	0.67 ± 0.18	−16.24 ± 0.50***	1.04 ± 0.24	−16.9 ± 0.50***
Anion gap (mmol/L)	15.12 ± 0.30	30.61 ± 0.48***	14.79 ± 0.16	30.26 ± 0.39***
Lactate (mmol/L)	1.84 ± 0.12	17.99 ± 055***	1.84 ± 0.12	18.69 ± 0.45***

Values are presented as mean ± SEM. Blood samples were collected before the test (at rest) and 3 min after the test (3 min). The data were analyzed using two-way ANOVA: two treatment (placebo and GTE) × two time points (at rest and 3 min after completion of the test). Time effect was observed for all the analyzed parameters (*p* < 0.001) with no treatment and time × treatment interactions (*p* > 0.05)

*** Significantly different (*p* < 0.0001) from the resting value (Bonferroni test)

Data regarding the parameters of exercise performance during RST are presented in Table 3. None of the parameters changed significantly after GTE supplementation, as compared to PL supplementation.

**Table 3:** Changes in the results of the repeated sprint test (4 × 15 s) in sprinters (*n* = 16) after 4-week supplementation with placebo (PL) or green tea extract (GTE)

	PL	GTE
Peak power^a^ (W/kg)	10.68 ± 0.12	10.86 ± 0.13
Mean power^a^ (W/kg)	8,91 ± 0.08	8.90 ± 0.09
Total work output^b^ (J/kg)	533.5 ± 4.89	533.8 ± 5.53
Fatigue index^a^ (%)	11.3 ± 1.07	12.2 ± 0.81

Values are presented as mean ± SEM. No significant differences were documented between treatments (paired *t* test)

^a^mean value for four bouts (4 × 15 s) of the exercise test

^b^sum of the values determined during four bouts (4 × 15 s) of the exercise test

A significant increase in resting total plasma polyphenol level (determined with Folin assay) was observed after GTE supplementation, when compared with the respective value recorded after administration of PL (388 ± 51.3 vs. 136 ± 11.3 µg GE/ml; *p* < 0.01).

The blood levels of oxidative stress and muscle damage markers are presented in Table 4. Significant time (*p* < 0.001), treatment (*p* < 0.01), and time × treatment interaction effects (*p* < 0.05) on TAC were observed. Only RST performed after PL supplementation caused an increase in TAC level at 5 min, which remained elevated after the 24-h recovery period (both changes were significant as compared to resting value). Similarly to the increase in plasma total polyphenols, elevation of resting plasma TAC was observed after GTE supplementation.

**Table 4:** Changes in blood parameters of oxidative stress and muscle damage induced by the repeated sprint test (4 × 15 s) in sprinters (*n* = 16) after 4-week supplementation with placebo (PL) or green tea extract (GTE)

	PL	GTE	Time	Treatment	Time × treatment
Total antioxidant capacity (TAC) (mmol/L)					
Rest	1.33 ± 0.04	1.52 ± 0.03^‡^	Yes	Yes	Yes
5 min	1.58 ± 0.04***	1.54 ± 0.04			
24 h	1.51 ± 0.05**	1.59 ± 0.06			
Uric acid (UA) (mg/dL)					
Rest	5.15 ± 0.26	4.31 ± 0.20	Yes	No	No
5 min	5.16 ± 0.26	4.30 ± 0.15			
24 h	6.61 ± 0.29***^, †††^	6.81 ± 0.28***^, †††^			
Albumin (AL) (g/dL)					
Rest	4.09 ± 0.14	4.06 ± 0.10	Yes	No	No
5 min	4.68 ± 0.18**	4.89 ± 0.20***			
24 h	4.20 ± 0.08^†^	4.44 ± 0.14^†^			
Superoxide dismutase (SOD) (U/g Hb)					
Rest	1,596.8 ± 55.7	1,524.0 ± 28.4	Yes	Yes	Yes
5 min	1,797.4 ± 33.9***	1,497.2 ± 32.5^‡‡‡^			
24 h	1,794.3 ± 34.3***	1,468.8 ± 25.8^‡‡‡^			
Glutathione peroxidase (GPx) (U/g Hb)					
Rest	46.8 ± 2.2	39.0 ± 2.1	No	No	No
5 min	38.8 ± 1.7	37.1 ± 1.1			
24 h	38.3 ± 3.2	37.8 ± 1.9			
Malondialdehyde (MDA) (µmol/L)					
Rest	1.15 ± 0.09	0.66 ± 0.04^‡^	Yes	Yes	No
5 min	1.48 ± 0.08**	0.85 ± 0.06^‡‡^			
24 h	0.86 ± 0.09^†††^	0.53 ± 0.02^††^			
Creatine kinase (CK) (U/L)					
Rest	192.0 ± 18.2	178.1 ± 15.0	Yes	No	No
5 min	219.5 ± 18.9	199.0 ± 17.2			
24 h	230.6 ± 21.5*	231.5 ± 19.8**			

Values are presented as mean ± SEM. Blood samples were collected before the test (at rest), 5 min after the test (5 min), and after 24 h of recovery (24 h). The data were analyzed using two-way ANOVA: two treatment (placebo and GTE) × three time points (at rest, 5 min, and 24 h after completion of the test)

*, **, *** Significantly different (*p* < 0.05; *p* < 0.01; *p* < 0.001, respectively) from the resting value (within the same treatment; Bonferroni test)

^††, †††^Significantly different (*p* < 0.01; *p* < 0.001, respectively) from the value determined at 5 min (within the same treatment; Bonferroni test)

^‡, ‡‡, ‡‡‡^Significantly different (*p* < 0.05; *p* < 0.01; *p* < 0.001, respectively) from the respective value determined after placebo treatment (Bonferroni test)

A similar pattern of post-RST changes in plasma UA and AL concentrations was noted after both GTE and PL supplementations (time effect, *p* < 0.001). Compared to values measured at rest and 5 min post-exercise, the increase in plasma UA was observed after 24 h of recovery. An increase in plasma AL was observed 5 min post-exercise (as compared to resting values), with subsequent decrease after 24 h of recovery (as compared to values at 5 min).

Significant time, treatment, and treatment × time interaction effects (each at *p* < 0.001) on erythrocyte SOD activity were noted. After the 4-week PL supplementation, the significant increase in erythrocyte SOD activity was documented 5 min post-exercise (as compared to respective value at rest). The analyzed parameter remained elevated (as compared to resting value) throughout the 24-h recovery period. No significant differences in resting SOD activity were documented between GTE and PL treatment. In turn, comparing to PL, GTE supplementation caused a decrease in SOD activity at 5 min and 24 h after RST. Contrary to SOD, no significant changes in GPx activity were noted, either in response to the RST (performed after both GTE and PL supplementations) or as a result of the 4-week GTE supplementation.

Both RST and GTE supplementations affected plasma concentration of MDA (*p* < 0.001 for both time and treatment effect). Similar to TAC value, plasma MDA increased 5 min after RST (as compared to resting value), but only after PL administration. However, after 24 h of recovery, a decrease in plasma MDA (as compared to level at 5 min) was observed after both PL and GTE treatments. As a result of GTE supplementation, a significant decrease in both resting and post-exercise (at 5 min) MDA levels was observed.

The RST, performed after both PL and GTE treatments, induced changes in plasma CK activity (time effect, *p* < 0.001). An increase in this parameter was observed after the 24-h recovery period as compared to resting value. No significant differences between PL and GTE treatments were documented at any of the three time points.

## Discussion

To the best of our knowledge, this study is the first one which analyzed the effect of GTE supplementation on oxidative stress in sprint-trained athletes. Our results suggest that supplementation with GTE prevents oxidative stress induced by high-intensity RST conducted in male sprinters.

Oxidative stress may be considered a condition in which the production of ROS exceeds the physiological capacity of the antioxidant system to render ROS inactive [2]. Previous studies showed that lipid radical production increases in response to short-term supramaximal anaerobic exercise [2, 33, 34]. In our study, RST performed after PL treatment enhanced the oxidative damage, since additional accumulation of lipid peroxidation by-products, expressed as plasma MDA, was noted 5 min post-exercise. However, increased plasma MDA, observed post-exercise, was followed by a decrease in plasma MDA during the 24-h recovery. According to some authors, adaptation to elevated hydroperoxide concentration, observed immediately after intense exercise, is associated with a concomitant increase in the antioxidant potential [35]. This acute adaptation includes both enzymatic [36] and non-enzymatic antioxidant systems [36, 37]. Thus, an increase in post-exercise plasma TAC observed in our athletes probably represented an adaptive response to the enhanced generation of ROS. Our finding is consistent with previous studies with the single 30-s Wingate test [35, 38].

Urate, proteins, and ascorbate are major antioxidants of human plasma [39]. Thus, the post-exercise increase in plasma TAC observed in our participants might be influenced, at least partially, by an increase in plasma AL concentration. Our findings are consistent with the results of a previous study [40], in which higher serum concentration of total AL was documented immediately after intense session of anaerobic exercise. Surprisingly, however, we observed the elevated levels of UA, a major contributor to plasma TAC, 24 h after RST rather than 5 min post-exercise.

The purine nucleotide system is extremely active during high-intensity exercise and muscle ischemic conditions. This is reflected by the release of hypoxanthine, ammonia, and UA into the circulation. Consequently, increased plasma UA documented in our athletes at 24 h after RST may reflect the enhancement of energy-rich purine phosphates catabolism and contribution of a XO to the free radical generating system [41]. On the other hand, although UA is the end product of the purine nucleotide degradation, it may be an important scavenger of free radicals in human plasma and in skeletal muscle during and after acute exercise [42]. This may be a potential reason for the lack of increase in plasma UA documented in our participants 5 min post-exercise, since circulating UA was postulated to be taken up by the recovering muscle, and serve as a free oxygen radical scavenger [43]. Baker et al. [38] reported a significant decrease in serum UA immediately after the 30-s Wingate test; this was followed by an increase in serum UA and concomitant decrease in serum lipid hydroperoxide and MDA concentrations 24 h post-exercise. Thus, elevated plasma levels of UA and TAC, observed during the recovery period of our study, were likely responsible for the attenuation of oxidative damage; this hypothesis is supported by decreased plasma MDA levels documented during the recovery period.

In the present study, RST performed after PL treatment caused a rise in erythrocyte SOD activity, in parallel to the above-mentioned reinforced non-enzymatic antioxidant potential of plasma. In another study [36], an increase in blood activity of the antioxidant enzymes (inter alia SOD and GPx) was noted in untrained men after a single boot of both maximal and submaximal exercises. However, we did not observe changes in erythrocyte GPx activity, which should be addressed in future studies.

There is some evidence that antioxidant supplementation could protect from exercise-induced muscular and oxidative damage, inflammation, muscle force loss, and fatigue [44]. This protection might accelerate recovery and, in turn, lead to increased exercise performance. In the present study, polyphenols of GTE contributed to an elevated resting plasma TAC. On the other hand, GTE supplementation prevented increase in post-exercise plasma TAC. Our observations are consistent with the results of another study [45], in which polyphenol-rich dark chocolate turned out to blunt the exercise-induced increase in plasma TAC. In our study, post-exercise plasma levels of TAC and MDA remained unchanged solely after GTE treatment. In contrast, an increase in both these parameters was observed 5 min after RST performed after PL treatment. These observations suggest that GTE supplementation may protect sprinters against oxidative stress induced by acute RST, by enhancing antioxidant defense at rest. In our previous study including previously untrained men who were subjected to a 4-week strength training [16], GTE supplementation protected against oxidative stress induced by acute muscular endurance test, as well as against muscular damage induced by the training alone. Similar observations were reported by Panza et al. [17], in a group of resistance-trained men. In both cited studies, significant decrease in post-exercise plasma CK activity was noted as a result of supplementation. However, in our present study, GTE supplementation provided no protection from exercise-induced muscle damage. On the other hand, a number of previous studies [6, 10, 46] revealed intensified muscle damage and hindered recovery as a result of antioxidant supplementation. Moreover, according to several authors [7, 9, 22], antioxidant supplementation may prevent some cellular adaptations to sport training. In view of our hereby presented findings, GTE intake is unlikely to affect sprint training adaptation, as resting SOD activity did not differ between PL and GTE treatments. In turn, the blunting effect of GTE supplementation on SOD activity post-exercise and after 24-h recovery was observed in our study, which strongly points to diminished production of superoxide anions [34]. Finally, green tea catechins were identified as a potential inhibitor of XO [25]. Although we did not measure plasma XO activity, no changes in plasma UA (the end product XO system) were noted after GTE supplementation. Recently, normal adaptations to exercise were observed in rats despite protection against oxidative stress resulting from long-term supplementation with large doses of antioxidant vitamins [12] or treatment with allopurinol as XO inhibitor [47]. Thus, our study showed that supplementation with GTE does not interfere with training adaptation of antioxidant enzyme system. Although GTE prevented exercise-induced oxidative stress, it neither modulated plasma CK activity nor affected the result of RST. This suggests that GTE supplementation neither protects against muscle damage nor improves repeated sprint performance in male sprinters.

The fact that the same athletes received both PL and GTE capsules represents a strength of the present study, especially in view of considerable individual variation in analyzed biomarkers. However, the fact that we used MDA, rather than more specific parameters recommended by EFSA [48], e.g., F_2_-isoprostanes, as a marker of lipid peroxidation can be potential limitation of our study. Similarly, determination of other antioxidants contributing to total antioxidant potential of the plasma, such as vitamin C or selected flavanols, could provide more accurate information on the response of antioxidant defense to acute exercise or GTE supplementation.

In conclusion, supplementation with GTE prevents oxidative stress induced by high-intensity repeated sprint test in male sprinters. On the other hand, neither protection from exercise-induced muscle damage, nor an improvement in sprint performance was noted after GTE intake. Our findings suggest that GTE intake is likely not beneficial in the case of sprinters, at least during the preparatory phase of their yearly training cycle. The effects of GTE supplementation during the competition phase of yearly training cycle, being associated with markedly greater exercise load, should be a subject of future research.
